# The Impact of Error-Consequence Severity on Cue Processing in Importance-Biased Prospective Memory

**DOI:** 10.1093/texcom/tgab056

**Published:** 2021-09-07

**Authors:** Kristina Krasich, Eva Gjorgieva, Samuel Murray, Shreya Bhatia, Myrthe Faber, Felipe De Brigard, Marty G Woldorff

**Affiliations:** Center for Cognitive Neuroscience, Duke Institute for Brain Sciences, Duke University, Durham, NC 27708, USA; Center for Cognitive Neuroscience, Duke Institute for Brain Sciences, Duke University, Durham, NC 27708, USA; Dept. of Psychology and Neuroscience, Duke University, Durham, NC 27708, USA; Dept. of Psychology and Neuroscience, Duke University, Durham, NC 27708, USA; Dept. de Psicología, Facultad de Ciencias Sociales, Universida de los Andes, Bogotá 111711, Columbia; Center for Cognitive Neuroscience, Duke Institute for Brain Sciences, Duke University, Durham, NC 27708, USA; Dept. of Communication and Cognition, Tilburg School of Humanities and Digital Sciences, Tilburg University, 5037 AB Tilburg, The Netherlands; Donders Institute for Brain, Cognition and Behaviour, Radboud University, 6525 EN Nijmegen, The Netherlands; Center for Cognitive Neuroscience, Duke Institute for Brain Sciences, Duke University, Durham, NC 27708, USA; Dept. of Psychology and Neuroscience, Duke University, Durham, NC 27708, USA; Dept. of Philosophy, Duke University, Durham, NC 27708, USA; Center for Cognitive Neuroscience, Duke Institute for Brain Sciences, Duke University, Durham, NC 27708, USA; Dept. of Psychology and Neuroscience, Duke University, Durham, NC 27708, USA; Dept. of Psychiatry, Duke University, Durham, NC 27708, USA

**Keywords:** cue recognition, EEG, parietal positivity, prospective memory, event-related potential

## Abstract

Prospective memory (PM) enables people to remember to complete important tasks in the future. Failing to do so can result in consequences of varying severity. Here, we investigated how PM error-consequence severity impacts the neural processing of relevant cues for triggering PM and the ramification of that processing on the associated prospective task performance. Participants role-played a cafeteria worker serving lunches to fictitious students and had to remember to deliver an alternative lunch to students (as PM cues) who would otherwise experience a moderate or severe aversive reaction. Scalp-recorded, event-related potential (ERP) measures showed that the early-latency *frontal positivity*, reflecting the perception-based neural responses to previously learned stimuli, did not differ between the severe versus moderate PM cues. In contrast, the longer-latency *parietal positivity*, thought to reflect full PM cue recognition and post-retrieval processes, was elicited earlier by the severe than the moderate PM cues. This faster instantiation of the parietal positivity to the severe-consequence PM cues was then followed by faster and more accurate behavioral responses. These findings indicate how the relative importance of a PM can be neurally instantiated in the form of enhanced and faster PM-cue recognition and processing and culminate into better PM.

## Introduction

People do not perform every intended task simultaneously. Instead, people rely on *prospective memory* (PM) to remember to perform intended tasks at the appropriate moment in the future (Meacham and Singer 1977). Successful PM operations require a dynamic coordination of numerous attention- and memory-related processes (Ellis 1996; Shelton and Scullin 2017), and *event-based PM* specifically relies on the recognition of an external cue that triggers PM recall and thus the associated PM task (Einstein and McDaniel 1990). Without successful PM cue recognition, event-based PMs would not be recalled reliably, and the prospective task may not be completed, resulting in a *PM error*.

The consequences following PM errors can be more or less severe depending on the relative importance of the prospective task. For example, the consequences of forgetting to buy medication will likely be more severe than the consequences of forgetting to buy apples. Surprisingly, though, there is not a strong empirical consensus of whether PM is better for more important than less important prospective tasks. Past studies of PM have manipulated the importance of the prospective task by introducing a prosocial and/or monetary incentive (e.g., Brandimonte et al. 2010; Cook et al. 2015; Rummel et al. 2017), emphasizing the relative importance of the PM over another non-PM task (e.g., Kliegel et al. 2001; Hering et al. 2014; Smith and Hunt 2014; Ball and Aschenbrenner 2018), or using emotionally valanced stimuli as PM cues (Clark-Foos et al. 2009; Altgassen et al. 2010; Rendell et al. 2011; May et al. 2012, 2015; Cona et al. 2015). Many of these studies showed faster and/or more accurate responses to important PM cues compared to less important ones (Altgassen et al. 2010; May et al. 2012, 2015; Hering et al. 2014; Cona et al. 2015; Cook et al. 2015; Ball and Aschenbrenner 2018). Others, however, failed to replicate this effect (Kliegel et al. 2001, 2004, Loft and Yeo 2007; Smith and Hunt 2014; Rummel et al. 2017) or found that increasing PM importance instead *impaired* responses to PM cues (Clark-Foos et al. 2009; Brandimonte et al. 2010; Brandimonte and Ferrante 2015). These mixed results are usually interpreted within the context of top–down motivation, strategic shifts in processing demands, and/or changes in bottom-up visual salience of arousing PM cues. Accordingly, there is still no clear framework that comprehensively accounts for the discrepant findings.

One possibility is that these accounts underemphasize the role of value-cognition interactions (Braver et al. 2014). That is, manipulating the importance of a PM might impact the cognitive and neural processing of PM cues beyond influences from top–down strategies and bottom-up influences. For instance, studies of attention have shown that manipulating reward- and/or punishment-associations changes the temporal dynamics of stimulus processing (Krebs et al. 2013; Schevernels et al. 2015) through different neural mechanisms than those for processing visually salient stimuli (Bachman et al. 2020). Some have further argued that value-based biases are cognitively (Awh, Belopolsky, and Theeuwes 2012; Failing and Theeuwes 2018) and neurally (MacLean and Giesbrecht 2015) unique from top–down or bottom–up biases. Similar value-cognition interactions have been observed in studies of working memory, cognitive control, and decision-making (e.g., Locke and Braver 2010; Maddox and Marman 2010; Pessoa and Engelmann 2010). Not accounting for the role of value-cognition interactions may be potentially problematic in studies of PM, where a traditional empirical approach is to present important PM cues in different experimental blocks as neutral or less-important PM cues. Different motivation and/or adopted task-strategies (e.g., strategic monitoring, Smith 2003; spontaneous retrieval, McDaniel et al. 2004) between the different blocks might obfuscate how importance uniquely biases the cognitive and neural processing of PM cues. The current study, therefore, sought to expand on current theories of PM by investigating how PM-task importance impacts processing of PM cues when top–down, blockwise strategies and processing demands as well as bottom–up stimulus valence are controlled. We further sought to investigate how these changes in processing would then ramify into different behavioral responses to PM cues as a function of the associated importance.

To address these questions, we used a novel paradigm designed to probe PM-cue processing while minimizing variability in top–down strategies and bottom–up visual processing demands. As set up by an *a priori* task narrative, participants assumed the role of a cafeteria lunch staff member serving lunches to fictional students (presented as face images). Most of the students were to receive a standard lunch, as denoted by a consistent button press on a game pad. A small percentage of the students (occurring as previously learned PM cues) had dietary restrictions and required one of two possible alternative lunches, each denoted by a different button press on the same game pad. Specifically, certain students required a dairy-free lunch to avoid a stomachache (moderate PM cues), whereas other students required a peanut-free alternative lunch to avoid a potentially life-threatening anaphylactic shock (severe PM cues). PM importance was thus manipulated by altering the associated consequence for committing a PM error. Critically, each experimental block contained both moderate and severe PM cues randomly interspersed to encourage similar experiment-block PM cue processing demands and task strategies.

The lunch-serving task required a high degree of vigilance to recognize the infrequent PM cues from perceptually similar non-PM cue stimuli as well as to marshal active control processing to override the frequent, repeated, behavioral response to students receiving the standard lunch. Accordingly, responses to the PM cues were expected to be slower and less accurate than responses to the non-PM face stimuli due to these additional attention and memory processes required on the PM-cue trials (Anderson et al. 2019). If responses to the severe PM cues turned out to be faster and/or more accurate compared to responses to the moderate PM cues, it would indicate that the relative importance of a PM, as defined here by the associated PM error-consequence, had biased prospective task performance. If, however, responses to severe and moderate PM cues did not differ, the findings would suggest that PM importance does not bias prospective task performance when top–down, block-wise strategies and processing demands as well as bottom–up stimulus valence are controlled.

To examine the cascade of neural processes underlying such behavioral effects, scalp-recorded electroencephalographic (EEG) and event-related potential (ERP) measures were collected while participants completed the lunch-serving task. Although there is some paradigm specificity to previously reported PM ERP effects (Bisiacchi et al. 2009; Cona et al. 2014; Cruz et al. 2016), past work has identified several cue-evoked ERPs as being associated with PM cue recognition, namely the *N300*, the early *frontal positivity*, and the longer-latency *parietal positivity* (see West 2011 for a review). The occipital-parietal *N300* and the sustained midline *frontal positivity* are neuroelectric responses—peaking about 250–400 ms after stimulus onset—that are commonly evoked by PM cues relative to perceptually dissimilar non-cues (West et al. 2001; West and Krompinger 2005; West et al. 2006). Perceptually similar non-PM cue lures, however, can also evoke an N300/frontal positivity (West et al. 2001; West and Covell 2001; West et al. 2003). Moreover, the N300 is typically not observed when PM cues are difficult to detect among non-PM cue stimuli, such as when the only defining feature of the cue is conceptual rather than perceptual (Wang et al. 2013; Wilson et al. 2013; Cousens et al. 2015; Cruz et al. 2016). Accordingly, both the N300 and frontal positivity likely reflect some initial recognition of potentially cue-relevant perceptual features and not full PM cue recognition *per se* (West 2011; Cousens et al. 2015).

More precise PM cue recognition seems instead to be reflected by the longer-latency sustained *parietal positivity*. This activity likely includes several sequential and partially overlapping ERP components, including the *P3*, the *parietal old-new memory effect*, and the *prospective positivity effect* (West 2011; Meier et al. 2014). The widely studied centroparietal P3 has been linked to the depth of cognitive analysis and context updating, particularly of infrequent targets (Donchin and Coles 1988; Kok 2001; Nieuwenhuis et al. 2005). The hallmark parietal old-new effect is an ERP positivity evoked by previously learned verses unlearned items and is thus reflective of recognition memory processes (Rugg and Nagy 1989; Rugg et al. 1998; Rugg and Curran 2007). The sustained prospective positivity is then thought to reflect PM cue recall as well as post-retrieval processes, such as the configuration and instantiation of the prospective task set (West and Wymbs 2004; Bisiacchi et al. 2009; West 2011). This neural component has been shown to be more pronounced for PM cues than for perceptually similar PM cue lures (West et al. 2001), even when the defining feature of the PM cue is conceptual rather than perceptual (Cousens et al. 2015; Cruz et al. 2016).

In the current work, the lunch-serving task consisted of perceptually similar, neutral face stimuli, where the distinguishing feature of the PM cue from other stimuli was primarily conceptual. Accordingly, a perceptually based N300 was not expected in response to the PM cues (Wang et al. 2013; Wilson et al. 2013; Cousens et al. 2015; Cruz et al. 2016). Instead, a frontal positivity was expected in response to PM cues relative to non-PM cues, which would reflect the initial detection of potentially relevant PM cue stimuli (West 2011; Cousens et al. 2015). If error-consequence severity impacts this initial detection, the frontal positivity was expected to be larger and/or to emerge earlier in response to the severe than to moderate PM cues. Such a finding would parallel one past study showing a larger frontal positivity in response to emotionally valanced PM cues compared to neutral ones (Cona et al. 2015). The frontal positivity, however, is thought to be mainly perception-driven (West et al. 2001; West and Covell 2001; West et al. 2003), and thus it might not be sensitive to variations in error-consequence severity across the perceptually similar PM cues in the current study. In addition, these different cue types appeared in separate blocks in such previous studies and thus could have resulted from different strategies or arousal levels between blocks.

Error-consequence severity may instead, or additionally, bias precise PM recognition from non-PM cue stimuli. If so, a larger and/or earlier parietal positivity would be expected in response to PM cues over non-PM cue stimuli, and an even larger and/or earlier parietal positivity would be expected in response to severe than to moderate PM cues. This idea parallels the findings from several studies showing that the P3 and the parietal old/new effect—constituents of the parietal positivity—were earlier in latency (Bachman et al. 2020) and/or larger in amplitude (Yeung and Sanfey 2004; Yeung et al. 2005; Elliott et al. 2020) in response to rewarding stimuli compared to not rewarding ones. Further, the parietal positivity has been shown to be greater in response to emotionally valent as opposed to emotionally neutral PM cues when these cues were in separate experimental blocks (Cona et al. 2015). The current work thus sought to expand on these previous findings by investigating whether PM error-consequence severity would also lead to an earlier and/or larger parietal positivity, thereby reflecting enhanced recognition and processing in the brain of more important PM cues. As such, the current work aimed at advancing understanding of how PM error-consequence severity impacts the cascade of cognitive and neural processes that support PM cue recognition and the subsequent prospective task performance.

## Materials and Methods

### Participants

Participants were 42 volunteers (*M*_age_ = 22 years, *SD*_age_ = 2.7 years, female = 22) recruited from the Duke University and the surrounding community. Participants were native English speakers, right-handed, with normal or corrected-to-normal vision, and no reported history of neurological or psychiatric disease. All participants provided written consent and were compensated $15/h for participating, in accordance with a protocol approved by Duke’s Institutional Review Board.

One participant was excluded due to overall poor performance on the lunch-serving task, as defined by a mean performance accuracy (across all trials) that was three standard deviations below the grand mean across all participants. Therefore, a total of 41 participants were included in the full analyses.

### Stimuli and Apparatus

**Figure 1:**
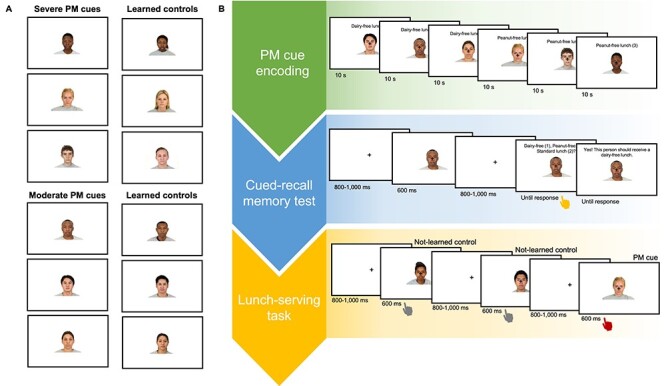
Experimental stimuli and procedures. (*A*) Severe and moderate PM cues and the learned controls that were roughly matched in gender and ethnicity. (*B*) Example experimental procedures for PM cue encoding, the cued-recall memory test, and the main lunch-serving task. During PM cue encoding, participants studied each PM cue for 10 s each. The order that participants learned each PM cue type was randomized, and the faces within each PM cue type were presented randomly. During the old/new cued-recall memory test, participants responded to face stimuli according to the designated lunch. The “new” face included in this test were supposed to receive a standard lunch and were also later embedded within the main lunch-serving task as learned control stimuli. Performance feedback was provided in the cued-recall memory task. Participants next completed an arithmetic distractor task (not pictured) that was used to disrupt the active retention of PM cues in working memory in order to target longer-term memory stores of the PM images during the main lunch-serving task. During the main lunch-serving task, participants responded to face stimuli via button press according to the required lunch (standard, dairy-free, or peanut-free). PM cues appeared in 10% of total trials and required a unique button response according to the specific PM cue type. Learned controls appeared in 10% of total trials and required a standard lunch. All other face stimuli were not previously learned and required a standard lunch. Performance feedback during the lunch-serving task was not provided.

The stimuli were sixty randomly selected colored images of neutral faces from the Chicago Face Database, including both male and female individuals of various ethnicities (Ma et al. 2015). Six faces were then randomly selected from this set of sixty to be PM cues and were loosely matched by gender and ethnicity with six other faces that would serve as learned controls (Fig. 1*A*; further details below). These PM cues and control stimuli were the same for all participants and experimental blocks. All stimuli were presented on a 24” LCD monitor with a screen refresh rate of 120 Hz.

Participants were seated approximately 80 cm away from the stimulus-presenting monitor in a dimly lit, electrically shielded room with no constraints on the head position. At this viewing distance, the face stimuli subtended a 6.28° x 5.03° visual angle. Stimulus presentation was controlled using Presentation 20.1 Software (Neurobehavioral Systems, Inc., Albany, CA), and all task responses were registered by key press on a Logitech gamepad.

### Procedures

The experimental procedures consisted of three main parts: encoding of the PM face-image stimuli, a working-memory distractor task, and the main lunch-serving task.

#### Task Narrative and PM Encoding

Participants first received detailed instructions for the lunch-serving task, which was introduced with the following narrative: “Welcome to your first day working at Central Senior High School! For your primary task, you will serve lunches to our high school students. A face will appear on the screen, and you will use buttons on the game pad to serve them lunch.” Participants were then instructed to deliver standard lunches to most of the students (denoted by a consistent button press), except for a select few students who required a unique lunch due to dietary restrictions (denoted by a unique button press). Participants’ failure to deliver the correct alternative lunch would result in moderate or severe health-related consequences for the students. Specifically, for students that required a dairy-free lunch, participants were told, “If you don’t [press the correct button], they will get a stomachache.” For students that required a peanut-free lunch, participants were told, “If you don’t [press the correct button], the student will die!” PM importance was thus manipulated by altering the associated consequence for failing to deliver an alternative lunch to these select students (i.e., PM errors), with dairy-free students operationalized as *moderate PM cues* and peanut-free students as *severe PM cues*.

Participants then studied each moderate and severe PM cue, the associated dietary restriction, and the required button response individually for 10 s each (Fig. 1*B*). The order that participants learned each PM cue type was randomized, and the faces within each PM cue type were presented randomly.

Participants next completed an “old/new” cued-recall memory test that was intended to encourage further PM cue encoding (Fig. 1*C*). Each trial sequence of the recognition task included a fixation stimulus (jittered 800–1000 ms), a face presentation (600 ms), another fixation stimulus (jittered 800–1000 ms), and a response prompt (until response). Participants were prompted to indicate, via a button press on a game pad, whether the student was to receive a dairy-free, peanut-free, or standard lunch. The “new” students included in this test were supposed to receive a standard lunch and would also be later embedded within the main lunch-serving task as students that required the standard lunch. Therefore, these *learned controls*—somewhat analogous to a PM cue lure or a retrospective memory cue—were learned at the same time as the PM cues but were not associated with a prospective task. They therefore required the same button response as all other not-learned, non-PM cue control stimuli that would be presented in the lunch-serving task.

Performance feedback on the recognition test was provided on each trial, and participants were required to respond correctly to each face four times to complete the test. There were five participants who only completed one round of the encoding procedures, but the other thirty-seven participants studied the faces and completed the old/new recognition test again to ensure adequate encoding of all PM cues and learned controls. EEG data were not recorded during these encoding procedures.

#### Distractor Task

Participants then completed a series of self-paced, multistep math problems for 30 s by indicating, with a button press, whether the answer was correct or incorrect (e.g., [5 × 7] + 8 = 43). This arithmetic distractor task was used to disrupt the active retention of PM cues in working memory to target longer-term memory stores of the PM images during the main lunch-serving task.

#### Lunch-Serving Task

Participants next completed the main lunch-serving task, which consisted of “delivering” standard lunches to most students and alternative lunches to the select students (moderate and severe PM cues) that were embedded in the face-image stimulus series. Each trial sequence began with a face presented for 600 ms followed by a jittered 800–1000-ms interstimulus interval (ISI), during which participants were to respond as to the appropriate lunch via a button press on a game pad (Fig. 1*D***)**. Participants were instructed to respond as quickly and accurately as possible. Performance feedback was not provided. Pseudo-randomly distributed thought probes appeared after half of the trials with PM cues and learned controls (i.e., after the jittered 800–1000 ms ISI). These probes measured the attentional focus of participants (i.e., focused or unfocused) for a separate empirical investigation not reported here.

The trials of the lunch-serving task were organized into 8 blocks, with 240 trials per block. Therefore, the 60 faces (48 not-learned controls, 6 learned controls, 3 moderate PM cues, and 3 severe PM cues) were each presented four times within a block. Therefore, there were 192 PM cue trials total (96 moderate PM cues, 96 severe PM cues) total across the entire experiment for each subject (12 of each type per block for 8 blocks). Each block contained both moderate and severe PM cues, at randomized points in the series.

After the fourth block, participants took a break from the lunch-serving task to view each PM cue for another 10 seconds each and to complete another recognition test. This served to refresh participants’ memory for the PM cues to minimize potential forgetting over time. Participants then completed the same 30-second arithmetic distractor task to disrupt working-memory maintenance before then resuming the lunch-serving task.

#### E‌EG Recording and Preprocessing

Online EEG data were recorded during the main lunch-serving task using a 64-channel, custom-layout, equidistant, extended-coverage cap **(**Woldorff et al. 2002**)** and an Actichamp amplifier from Brain Products (BrainVision, Morrisville, NC, USA) with active electrodes (actiCAP). One electrode was positioned below the left eye to record the vertical electrooculogram (EOG), and two electrodes were placed lateral to the outer canthi of the two eyes to measure the horizontal EOG. The electrodes are reported with respect to the extended 10–10 system naming convention. Our custom montage, however, does not overlap perfectly with the 10–10 montage, so electrode sites are reported as those that are closest to the 10–10 montage. All the electrode locations were less than ~1 cm from the cited standard 10–10 site.

The impedances of the ground, left mastoid, and right mastoid electrodes were maintained below 5 kOhms, with the rest of the electrodes maintained below ~15 kOhms. Data were referenced online to the right mastoid and re-referenced offline to the algebraic average of the left and right mastoids. Data were digitized at a 500-Hz sampling rate per channel using a three-stage cascaded integrator-comb filter with a corner frequency of 130 Hz. Then, offline, data were filtered with a low-pass filter (40 Hz cutoff), downsampled to 250 Hz, and further filtered with a high-pass filter (.05 Hz cutoff) using the EEGLab software package (La Jolla, CA).

The data were then epoched from −600 to 2000 ms relative to each face-stimulus onset and then baseline corrected from −200 to 0 ms relative to that onset. Channels with excessive noise artifacts were interpolated based on surrounding electrodes using a spherical-spline procedure (Perrin et al. 1989). To detect eye movements and blinks that occurred directly prior to or during the face stimulus presentation, data were submitted to an algorithm using a 200-ms wide window moving across the epoch from −100 to 600 ms in 50-ms steps. Epochs with peak-to-peak voltage differences exceeding 24 μV in the corresponding EOG channels were marked for rejection. Eye blinks that occurred at any point in the epoch were corrected with independent components analysis (ICA), with no more than two components removed for this correction. After ICA, data were again submitted to a step function algorithm using a 200-ms wide window moving across the epoch from −100 to 1600 ms in 50-ms steps to identify for rejection any trials with eye blinks that were not successfully corrected for with the ICA procedures. Epochs were also excluded if they contained voltage artifacts that were greater than +/− 100 μV in any channel during the face stimulus presentation. Across all subjects, there were of 16% of total epochs excluded due these blink and eye movement-related voltage artifacts.

The two epochs following each trial that contained a thought probe were excluded to limit any contribution from additional cognitive processes incurred following thought probe (20% of total epochs). Epochs with no behavioral response or that had behavioral response times faster than 200 ms were also excluded (3% of total epochs). In total, 64% of all epochs were included in the analyses after all exclusionary criteria were satisfied.

ERPs were time-locked to the onset of the face stimuli and selectively averaged using the Fieldtrip toolbox (Oostenveld et al. 2011).

#### Data Analysis

Behavioral task accuracy for the main lunch-serving task was computed as the proportion of correct button presses, and response times (RTs) were recorded relative to the face stimulus onset. These behavioral measures were then compared across conditions following a hierarchical approach. First, accuracy and RT (correct trials only) for the not-learned controls—which provide a baseline measure of non-PM cue stimulus processing—and for the learned controls—which provide a baseline measure of processing for previously learned stimuli without an associated PM intention—were compared to assess changes in performance specific to having previously learned the stimuli. Accuracy and RT (correct trials only) for the learned controls were then compared with the PM cues (moderate and severe PM cues averaged together; correct trials only) to assess changes in performance that were specific to accurate PM-cue recognition beyond the effects of learned versus unlearned stimuli. Critical to the primary research question, accuracy for the moderate and severe PM cues were compared to gauge the impact of error-consequence severity on successful PM. The RTs for severe and moderate PM cues were analyzed using a 2 (consequence: moderate or severe) × 2 (correctness: correct or incorrect) repeated-measures ANOVA to further investigate the impact of PM consequence severity on behavioral performance. As such, there were multiple overlapping comparisons used to investigate accuracy (*m* = 3: not-learned vs learned controls; learned controls vs PM cues; severe vs moderate PM cues) and RT (*m* = 6: not-learned vs learned controls; learned controls vs PM cues; severe vs moderate PM cues correct trials; severe vs moderate PM cues incorrect trials; moderate PM cues correct vs incorrect trials; severe PM cues correct vs incorrect trials). Holm-adjusted *P*-values were thus used to conservatively correct for family-wise error rates for each of these analyses (Holm 1979). The null hypothesis was then rejected when *p_Holm_* < 0.05.

EEG data were analyzed from stimulus onset to 1.0-s poststimulus onset at the participant level using cluster-based nonparametric permutation testing (Maris and Oostenveld 2007). Significant clusters were identified as consecutive time points and adjacent electrode channels that showed similarly significant differences between the two conditions—where the *t*-statistic exceeded a threshold of *p* < 0.05. A minimum of three neighboring channels were required for the selected sample to be included in the clustering algorithm. The cluster-level statistic was defined as the sum of the *t*-values within a cluster, and the maximum cluster-level statistic was used as the test statistic to evaluate the effect of the experimental conditions being tested. A permutation analysis—examining random permutations of the two conditions to estimate a null distribution—was then conducted to assess whether the test statistic was larger than chance. The reference distribution was estimated using the Monte Carlo method from 10,000 random sets of permutations, with the test statistic computed for each random partition as the maximum of the cluster-level summed *t*-values. The proportion of cluster-level statistics from the random partitions that exceeded the observed maximum cluster-level statistic was used as the estimated *p*-value (α = 0.025) for that cluster.

In total, there were four cluster-based permutation analyses conducted in the current study: one comparing not-learned and learned controls (correct trials only), one comparing learned controls and PM cues (correct trials only), one comparing moderate and severe PM cues within correct trials only, and one comparing moderate and severe PM cues within incorrect trials only.

While a cluster-based permutation analysis corrects for the multiple comparisons made across all the time points within a single analysis, the estimated *p*-value for each identified cluster does not account for when the same dependent variables are analyzed across multiple cluster-based permutation analyses as was the case with our hierarchal approach. Therefore, to conservatively correct for these multiple comparisons, we Holm-adjusted the estimated *p*-values for all identified clusters across the four cluster-based permutation analyses (*m* = 27). Effects were then considered significant when the estimated *p_Holm_* < 0.025. The complete findings from these analyses are reported in the Supplementary Appendix.

If the significant effects appeared temporally consistent with the onset of specific components, we also computed the average onset latency for the elicited ERP components using the 50% amplitude criterion (Luck 2014), which entails finding the point in time where the component amplitude reached 50% of its maximum amplitude (using the *latency* function from Liesefeld 2018), all relative to the pre-cue baseline. This is because cluster-based permutation tests do not provide precise estimation of the temporal onset of significant clusters (Sassenhagen and Draschkow 2019). In this way, our approach afforded insights into both amplitude and latency differences in the neural responses to the difference task stimuli and conditions.

## Results

### PM Cued-Recall Memory Test Performance at Encoding and at Refreshing

We first investigated the behavioral performance accuracy on the initial old/new cued-recall memory test to assess whether there were any differences in encoding across the different PM cues and learned controls. Performance accuracy was computed as the proportion of correct trials. A one-way repeated measures ANOVA showed that accuracy was not statistically different across the severe PM cues (*M* = 0.91, *SE* = 0.02), moderate PM cues (*M* = 0.89, *SE* = 0.02), and learned controls (*M* = 0.91, *SE* = 0.02); *F*(2,80) = 1.86, *p* = 0.163, *η_p_^2^* = 0.04. Likewise, a one-way repeated measures ANOVA showed that performance accuracy on the old/new recognition test that was used to refresh the PM-cue encoding half-way through the main lunch-serving test did not differ across the severe PM cues (*M* = 0.96, *SE* = 0.01), moderate PM cues (*M* = 0.94, *SE* = 0.01), and learned controls (*M* = 0.95, *SE* = 0.01); *F*(2,80) = 1.13, *p* = 0.328, *η_p_^2^* = 0.03. These collective findings indicate that there was no difference in how well the different PM cue types and learned controls were initially encoded or retained within memory and thus cannot account for any importance-biases in PM cue recognition that might be observed.

### The Effects of Previous Learning

**Figure 2:**
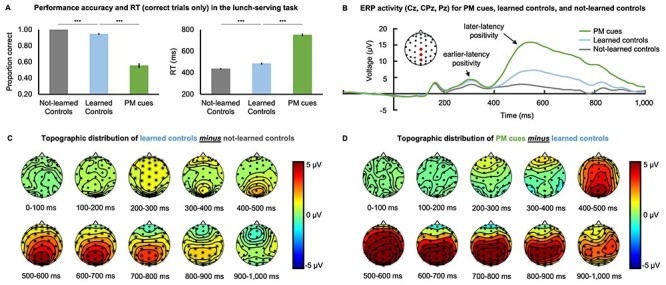
(*A*) Performance accuracy and RT (correct only trials) for the not-learned controls, learned controls, and PM cues. Asterisks indicate *p_Holm_* < 0.05 (^*^) or *p_Holm_* < 0.001 (^*^^*^^*^). Error-bars represent standard errors. (*B*) ERP traces of the not-learned controls, learned controls, and PM cues averaged over the Cz, CPz, and Pz electrode sites, showing a similarly larger early-latency positivity for all learned stimuli (PM cues and learned controls) relative to the not-learned controls, followed by a larger later-latency positivity for learned controls, which was larger still for PM cues. (*C*) The topographic distribution of the learned controls minus not-learned controls contrast, showing the fronto-central distribution of the enhanced early positivity, followed by the larger centroparietally distributed longer-latency positivity. Asterisks (^*^) indicate neighboring electrode channels where the cluster estimated *p_Holm_* < 0.025. (*D*) The topographic distribution of the PM cues minus learned controls contrast, showing the still further enhanced fronto-central longer-latency parietal positivity. Asterisks (^*^) indicate neighboring electrode channels where the cluster estimated *p_Holm_* < 0.025.

Before investigating our main research question regarding the effects of error-consequence severity, we first established the common behavioral and neural patterns of the PM processes in the novel lunch-serving paradigm used here. Performance accuracy on the main lunch-serving task, as measured by the proportion of correct trials, was first compared between the not-learned and learned controls to assess how previously learning the face stimuli in general might impact performance. Accuracy for these different controls was expected to be near ceiling because they both required the same, automatic, default behavioral response without the demanding active control processing required for remapping to a different motor response. As illustrated in Figure 2*A*, accuracy was indeed near ceiling for both the not-learned (*M* = 0.997 correct, *SE* = 0.001 correct) and learned controls (*M* = 0.95 correct, *SE* = 0.01 correct). That said, a paired-samples *t*-test did show significantly better accuracy for not-learned relative to learned control stimuli, *t*(40) = 4.53, *p_Holm_* < 0.001, *d* = 0.71. Within correct trials, RTs to the not-learned controls (*M* = 436 ms, *SE* = 7 ms) were also significantly faster than RTs to the learned controls *(M* = 484 ms, *SE* = 10 ms), *t*(40) = −8.53, *p_Holm_* < 0.001, *d* = −1.33. Together, these findings indicate a slight performance cost associated with previously learned controls, likely due to additional recognition processes triggered from previous learning.

Supporting this interpretation, a nonparametric cluster-based permutation analysis of the neural responses (applied to correct trials only) showed significant differences between the not-learned and learned controls, as illustrated in Figure 2*A* and *B*. These differences were characterized by a larger early-latency frontal-central positivity (*p_Holm_* < 0.001) as well as a larger longer-latency centroparietal positivity for learned controls compared to the not-learned controls (*p_Holm_* = 0.001). These findings thus indicate modulations of the stimulus-evoked neural responses due to the previous learning in this paradigm.

### The Effects of PM

Learned controls were next compared with the PM cues (averaged across moderate and severe PM cues) to investigate the effects of PM cues beyond previous learning. A paired-samples *t*-test showed that accuracy for the learned controls (*M* = 0.95 correct, *SE* = 0.01 correct) was substantially better than accuracy for the PM cues (*M* = 0.56 correct, *SE* = 0.03 correct), *t*(40) = 12.19, *p_Holm_* < 0.001, *d* = 1.90. The RTs for learned controls *(M* = 484 ms, *SE* = 10 ms) were also much faster than RTs for PM cues (*M* = 747 ms, *SE* = 12 ms), *t*(40) = −16.91, *p* < 0.001, *d* = −2.64. Together, these findings show a prospective task performance decrement, likely because of the additional neural and cognitive processes that are required for PM cue recognition, PM recall, and post-retrieval processes as well as processes involved in the preparation and execution of different motor responses.

The ERP activity patterns reflected the cascade of neural responses that led to the observed behavioral responses. As illustrated in Figure 2*B* and *D*, the cluster-based permutation analysis (correct trials only) showed that the early-latency frontal-central positivity evoked by the learned controls (relative to the unlearned controls) did not statistically differ from activity evoked by the learned PM cues (*p_Holm_* = 0.454). This is consistent with previous accounts showing that both PM cues and perceptually similar non-PM stimuli evoke a frontal positivity that reflects the early detection of familiar (previously learned) cue-relevant features (West et al. 2001; West and Covell 2001; West et al. 2003; West and Krompinger 2005; West et al. 2006). As predicted, there was no cue-evoked N300 observed in the current study, likely because of the similar perceptual features of the PM cues and the other task stimuli (West et al. 2003; Wang et al. 2013; Wilson et al. 2013; Cousens et al. 2015; Cruz et al. 2016).

The cluster-based permutation analysis did, however, show a significant difference in neural responses that was characterized by a large, longer-latency, centroparietal positivity for PM cues compared to learned controls (*p_Holm_* = 0.003). This effect was temporally and spatially consistent with the parietal positivity that has been reported to be evoked by PM cues, albeit the positivity observed here also extended to over somewhat more anterior scalp regions. Past research has previously linked this parietal positivity with full PM cue recognition as well as with PM recall and post-retrieval PM processes (West 2011).

Considering the above collective findings, the current study, using a novel PM paradigm, showed a behavioral performance cost for previously learned stimuli, which was substantially greater for PM cue trials. Further, the typical neural responses associated with PM cue recognition were observed, namely the frontal positivity likely reflecting the initial recognition of previously learned potentially cue-like features (West 2011; Cousens et al. 2015) and the subsequent PM-specific parietal positivity that has been previously linked to full PM cue recognition, PM recall, and post-retrieval processes (Bisiacchi et al. 2009; West 2011).

### The Effects of Error-Consequence Severity on PM

**Figure 3:**
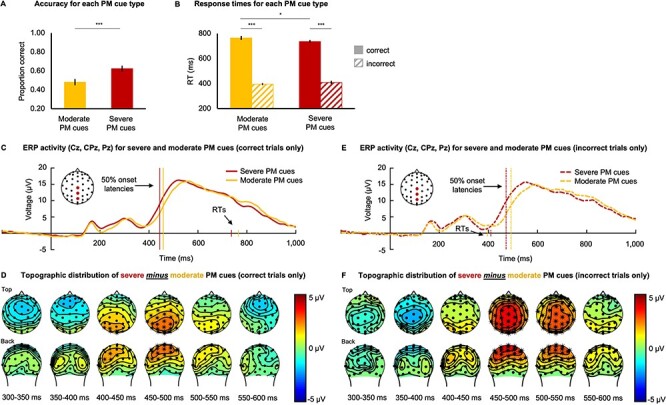
(*A*) Performance accuracy for the severe and moderate PM cues. Performance was more accurate for severe PM cues than moderate PM cues. (*B*) Responses times (RT) for severe and moderate PM cues. RTs were much slower for correct verses incorrect trials, although they were faster for correctly identified severe than moderate PM cues. In contrast, for incorrect trials there was no difference in response times between severe and moderate PM cues. For panels A and B, asterisks indicate *p_Holm_* < 0.05 (^*^) or *p_Holm_* < 0.001 (^*^^*^^*^). Error-bars represent standard errors. (*C*) ERP traces of the severe and moderate PM cues averaged over Cz, CPz, and Pz channels for correct trials only. The large vertical lines indicate the average 50% onset latency for each PM cue type. The short vertical lines indicate the average RT for each cue type. There was a significant difference in the parietal positivity that was characterized by an earlier onset latency for severe than moderate PM cues. (*D*) The topographic distribution of the severe minus moderate PM cues contrast for correct trials only showing a difference in the parietal positivity activity, resulting from the earlier onset latency for severe than PM cues. Asterisks (^*^) indicate neighboring electrode channels where the cluster estimated *p_Holm_* < 0.025. (*E*) ERP traces of the severe and moderate PM cues averaged over Cz, CPz, and Pz channels for incorrect trials only. There was a significant difference in the parietal positivity that was characterized by an earlier onset latency for severe than PM cues. On these incorrect trials, however, the neural response differences followed the behavioral responses, for which the RTs did not differ for severe vs. moderate PM cues. (*F*) The topographic distribution of the severe minus moderate PM cues contrast for incorrect trials only, showing increased parietal positivity for the severe due to the earlier onset for that condition. Asterisks (^*^) indicate neighboring electrode channels where the cluster estimated *p_Holm_* < 0.025.

Critical to our main research question, we next assessed how error-consequence severity for the two PM cue types impacted prospective task performance and the corresponding neural responses elicited by those cues. As illustrated in Figure 3*A*, a paired-samples *t*-test showed that accuracy for severe PM cues (*M* = 0.62, *SE* = 0.03) was significantly greater than accuracy for moderate PM cues (*M* = 0.48, *SE* = 0.03), *t*(40) = 7.00, *p_Holm_* < 0.001, *d* = 1.09. This finding indicates that an increased associated error-consequence severity also resulted in higher PM accuracy.

RTs were next examined using a 2 (error-consequence severity: moderate or severe) × 2 (correctness: correct or incorrect) repeated-measures ANOVA. Illustrated in Figure 3*B*, the findings showed a large main effect of correctness, with correct trials (*M* = 751 ms, *SE* = 11 ms) being much slower than incorrect trials [(*M* = 402 ms, *SE* = 11 ms), *F*(1,40) = 630.35, *P* < 0.001, *η_p_^2^* = 0.94] (Fig. 3), and pairwise comparisons showed that this difference occurred for both moderate PM cues, *t(*40) = 22.20, *p_Holm_* < 0.001, *d* = 3.47, and severe PM cues, *t(*40) = 22.22, *p_Holm_* < 0.001, *d* = 3.47. This suggests that there was a major difference in whether PM-supporting attention and memory processes were engaged prior to the rapid behavioral responses being initiated on incorrect trials relative to those engaged on correct trials.

Although there was not a significant main effect of error-consequence severity on RT, *F*(1,40) = 0.83, *p* = 0.369, *η_p_^2^* = 0.02, there was a significant error-consequence severity by correctness interaction, *F*(1,40) = 8.11, *P* = 0.007, *η_p_^2^* = 0.17. Pairwise comparisons indicated that, within correct trials, RTs for severe PM cues (*M* = 737 ms, *SE* = 12 ms) were significantly faster than RTs for moderate PM cues (*M* = 765 ms, *SE* = 16 ms), *t*(40) = −2.34, *p_Holm_* = 0.023, *d* = −.37. In contrast, for the much faster responses on incorrect PM-cue trials, the RTs for severe (*M* = 410 ms, *SE* = 12 ms) and moderate (*M* = 396 ms, *SE* = 9 ms) PM cues did *not* statistically differ from each other, *t(*40) = 1.39, *p_Holm_* = 0.172, *d* = 0.22. These collective findings further suggest that the PM-specific processes were not engaged prior to the very fast behavioral responses on the incorrect trials, but which were engaged—appearing to be significantly biased in favor of higher-valued cues—prior to the much later behavioral responses on the correct trials.

The corresponding cue-evoked neural responses were next analyzed to examine the neural processes that led to these behavioral response differences. First, we compared cue-evoked responses to severe and moderate PM cues for correct trials only. Illustrated in Fig. 3*C* and *D*, a cluster-based permutation analysis showed that the only significant difference was characterized by a greater parietal positivity for the severe compared to moderate PM cues (*p_Holm_* = 0.003), a difference that seemed to emerge at the onset of the parietal positivity. To test this idea, we assessed differences in the 50% onset of the parietal positivity between the severe and moderate cues. A 348 to 700-ms time window was used to isolate the relative time frame of the parietal positivity while avoiding conflation with adjacent components. A paired-samples *t*-test indeed showed that the 50% onset latency for this component occurred significantly earlier for severe PM cues (*M* = 444 ms, *SE* = 5 ms) relative to moderate PM cues (*M* = 458 ms, *SE* = 6 ms), *t*(40) = −3.12, *p_Holm_* = 0.022, *d* = −.49, indicating that the parietal positivity did indeed occur significantly earlier for the higher-valued compared to lesser-valued PM cues. Of note, illustrated in Figure 3*C*, this earlier onset latency paralleled—but preceded—the behavioral-response RT differences observed for severe relative to moderate PM cues when the response button was later pressed. This importantly suggests that the earlier PM cue recognition processing for the severe versus moderate PM cues contributed to the earlier accurate behavioral response for the severe PM cue.

We next compared neural responses to severe and moderate PM cues in the incorrect trials. Of the 2,907 total incorrect PM cue trials, participants mistakenly delivered the standard lunch 95% of the time, whereas participants only delivered the incorrect alternative lunch in only 5% of those trials. Therefore, in most incorrect trials, it was likely that PM-specific processes were not fully engaged to marshal active control processing to inhibit the frequent, repeated, standard-lunch-behavioral response. This would result in a very fast—but incorrect—default response. Further supporting this idea, findings from a cluster-based permutation analysis, illustrated in Figure 3*E* and *F*, showed that the only significant difference between severe and moderate PM cues in the incorrect trials was again observed within the partial positivity (*p_Holm_* = 0.003). We next computed the average 50% onset latency of the parietal positivity for each PM cue type, and a paired-samples *t*-test showed that the onset latency was again earlier for the severe (*M* = 472 ms, *SE* = 7 ms) than moderate (*M* = 492 ms, *SE* = 6 ms) PM cues, *t*(40) = −2.59, *p_Holm_* = 0.022, *d* = −.40. Collectively, this suggests that PM cue recognition did occur even on incorrect trials and that this recognition occurred earlier for the higher-valued compared to lesser-valued PM cues. Importantly, however, and as illustrated in Figure 3*E*, both average onset latencies for this neural response occurred *after* the corresponding average RT, suggesting that full PM cue recognition did not occur prior to participants initiating the very fast behavioral responses on the incorrect trials, and indeed that the neural recognition of the PM cue importance on these trials did not actually occur until after the behavioral response.

## General Discussion

The current study was aimed at advancing understanding of importance-biased PM by investigating how error-consequence severity impacts the cascade of cognitive and neural processes that support PM cue processing and the subsequent prospective task performance when top-down, block-wise strategies as well as bottom-up stimulus valence are controlled. Within a novel PM paradigm that integrated a narrative of a possible real-life scenario, participants completed a virtual lunch-serving task that required a standard default response for non-cues—either learned (infrequent) or not learned (frequent) control stimuli—along with infrequent unique responses to previously learned PM cues. These PM cues could be of either high or low importance, as defined by the relative severity of the consequences of committing a PM error in the response to them. Importantly, these two PM cue types were interspersed randomly across all experimental blocks to encourage the same task strategy, processing demands, and sustained arousal (e.g., strategic monitoring, Smith 2003; spontaneous retrieval, McDaniel et al. 2004), and to focus instead on reactive processes upon the occurrence and recognition of a PM cue and its importance/error-consequence severity. Scalp-recorded EEG revealed how the neural processes underlying PM cue recognition varied by relative error-consequence severity as well as how these modulations related to later prospective task performance.

Because we used a novel PM paradigm, we first established the behavioral and neural signatures of PM cue recognition and subsequent behavioral performance. We found slower and less accurate behavioral responses for previously learned stimuli, with an even greater performance decrement for PM cues. The EEG evidence suggests that these performance costs were inversely related to the extent to which attention and memory processes were invoked. That is, the previously learned controls showed a greater early-latency positivity relative to not-learned controls, which was spatially and temporally consistent with the *frontal positivity* that is thought to reflect the initial detection of potentially cue-relevant features (West 2011; Cousens et al. 2015). The frontal positivity has been reported to be evoked by both PM cues and perceptually similar non-cues (West et al. 2001; West and Covell 2001; West et al. 2003), and indeed the PM cues here also showed a cue-evoked frontal positivity that did not statistically differ from learned controls. At longer latencies, the learned controls showed a greater centroparietal positivity relative to the not-learned controls, and the PM cues showed a still greater positivity over the learned controls. This longer-latency positivity was spatially and temporally consistent with the parietal positivity*,* which has been previously observed in response to PM cues and which is thought to consist of several sequential and partially overlapping ERP components—namely the infrequent-target-processing *P3*, the recognition memory parietal old-new effect, and the PM-specific prospective positivity.

Critical to our main research question regarding error-consequence severity, behavioral responses were faster and more accurate for the PM cues associated with a severe error consequence compared to those associated with only a moderate error consequence. This finding indicates that increasing the error-consequence severity serves to increase the relative importance of the PM, and that PM is biased in favor of more important PM even when top–down, block-wise strategies and processing demands as well as bottom-up stimulus valence are controlled. The EEG evidence suggests that this importance-biased PM emerges at least in part from earlier full PM cue recognition. Supporting this idea, there was no observed difference in the early-latency frontal positivity between the severe and moderate PM cues, despite the differences in performance accuracy. Although, this finding deviates from past research showing differences in the frontal positivity across emotionally valanced and emotionally neural PM cues (Cona et al. 2015), it does bolster the idea that the frontal positivity is particularly sensitive to perceptual features of PM cues, rather than the conceptual ones. Moreover, the faster and more accurate responses for severe PM cues relative to moderate PM cues here did not appear to emerge from differences in the initial detection of potentially relevant PM cue-like features.

Instead, correctly identified severe PM cues showed an earlier onset of the somewhat longer-latency parietal positivity compared to correctly identified moderate PM cues, suggesting that full cue recognition as well as the associated post-retrieval processes occurred earlier for the more important PM cues. Further, this earlier parietal positivity paralleled—but preceded—the faster RTs of the behavioral responses for severe than moderate PM cues, with these neural differences occurring several hundred milliseconds before those behavioral responses. This suggests that earlier cue recognition and post-retrieval processes for severe PM cues contributed to the faster (accurate) behavioral responses that followed. Interestingly, on incorrect trials, which had much shorter RTs than did correct trials, a similar difference in the onset latency of the parietal positivity was observed between severe and moderate PM cues, but in this case occurring well after those behavioral responses. This suggests that full cue recognition, including the distinguishing of their severity/importance, did occur even on the incorrect trials and that this recognition was still biased in favor of the more important cues. Importantly, however, the onset latencies of the parietal positivity, including their differential onset as a function of severity/importance, occurred much later than the RTs on the incorrect trials, which did not differ for severe versus moderate PM cues. Such a pattern thus suggests that on those trials, the (incorrect) behavioral response was initiated and executed before full cue recognition occurred.

The current study cannot disambiguate which specific component within the parietal positivity accounts for the earlier onset latency for severe than for moderate PM cues. That said, converging evidence suggests that an earlier P3 may best account for this finding. Specifically, the P3 is the earliest component observed within the parietal positivity (West 2011), and it has been previously been shown to occur earlier in response to rewarding stimuli, albeit not in the context of PM (Bachman et al. 2020). It is also possible that the parietal old/new effect was accelerated in response to severe than moderate PM cues, given that it is tightly linked to recognition memory (Friedman and Johnson Jr 2000; Mecklinger 2000) and sensitive to variations in reward (Elliott et al. 2020). This speculation aligns with the idea that faster cue recognition is a critical process that underlies the behavioral advantage for more important PM cues when importance is conceptually defined. Regardless, if the earlier onset of the parietal positivity is due to an earlier onset of the P3 or of the parietal old/new effect (or both), it may be that the sequelae of such an effect could be an earlier onsetting of the prospective positivity that presumably followed within the parietal positivity complex. Future research is therefore necessary to determine whether and how these various postcue recognition processes are uniquely sensitive to variations in value.

Participants were instructed to respond to all task stimuli both quickly and accurately, such that neither was emphasized over the other. It is possible, though, that some participants—as a global task strategy—might have prioritized speed over accuracy despite the task instructions, and that these participants were overall faster but less accurate than participants who did not choose such a strategy (and the reverse true for those who may have prioritized accuracy over speed). This possible between-subjects effect, however, cannot account for the within-subject behavioral advantage for learned controls over PM cues because each stimulus type occurred randomly within all experimental blocks, and responses were both faster and more accurate for learned controls over PM cues. A speed/accuracy trade-off also cannot account for the within-subject behavioral advantage for severe over moderate PM cues because each PM cue type also occurred randomly within all experimental blocks, and responses were both faster and more accurate for severe than moderate cues.

In summary, the current work investigated the cascade of cognitive and neural processes underlying the recognition of PM cues as a function of their importance, as defined by the associated PM error-consequence severity, as well as their corresponding effects on prospective task performance. The findings suggest that faster and more accurate prospective task performance for important PMs can be partially attributed to a faster recognition of more important versus less important PM cues, even when top-down, block-wise strategies and processing demands as well as bottom-up stimulus valence are held constant. In contrast, the still earlier neural activity reflecting recognition of potentially cue-relevant perceptual features (the frontal positivity) was *not* sensitive to variations in value. The current work contributes to theories of PM that have largely not considered the role of value-cognition interactions. Specifically, it shows that the relative importance of PM can quicken PM cue recognition and processing, which then ramifies into quicker behavioral responses to such cues. Accordingly, the current work shows that processes reflecting neural and cognitive biases associated with PM cue recognition are important contributing factors to value-based biases in PM.

## Funding

This project was made possible through the support of a grant from the John Templeton Foundation. The opinions expressed in this publication are those of the authors and do not necessarily reflect the views of the John Templeton Foundation.

## Notes

Portions of this work have been previously presented at the Annual Meeting of the Cognitive Neuroscience Society.

Portions of this work contributed to a Senior Thesis by SB under the primary direction of MGW at Duke University, with KK, EG, and FDB also serving as members of the thesis committee.

KK, SM, and MF conceptualized and refined the initial research idea. KK, EG, and MGW further refined the research idea, designed, tested, and iteratively refined the experimental paradigm. KK, EG, and SB performed data collection and analyses under the guidance of MGW. KK, EG, SM, SB, MF, FDB, and MGW wrote and edited the culminating manuscript. *Conflict of Interest*: All authors declare no significant competing financial, professional, or personal interests that might have influenced the performance or presentation of the work described in this manuscript.

## Supplementary Material

Krasich_CerebralCortex_appendix_tgab056

## References

[ref1] Altgassen M, Phillips LH, Henry JD, Rendell PG, Kliegel M. 2010. Emotional target cues eliminate age differences in prospective memory. Q J Exp Psychol. 63(6):1057–1064. 10.1080/17470211003770920 20401810

[ref2] Anderson FT, Strube MJ, McDaniel MA. 2019. Toward a better understanding of costs in prospective memory: A meta-analytic review. Psychol Bull. 145(11):1053. 31464456 10.1037/bul0000208

[ref2a] Awh E, Belopolsky AV, Theeuwes J. 2012. Top-down versus bottom-up attentional control: A failed theoretical dichotomy. Trends Cogn Sci. 16(8):437–443. 22795563 10.1016/j.tics.2012.06.010 PMC3426354

[ref3] Bachman MD, Wang L, Gamble ML, Woldorff MG. 2020. Physical salience and value-driven salience operate through different neural mechanisms to enhance attentional selection. J Neurosci. 40(28):5455–5464. 32471878 10.1523/JNEUROSCI.1198-19.2020 PMC7343324

[ref4] Ball BH, Aschenbrenner AJ. 2018. The importance of age-related differences in prospective memory: Evidence from diffusion model analyses. Psychon Bull Rev. 25(3):1114–1122. 28600714 10.3758/s13423-017-1318-4 PMC5796868

[ref5] Bisiacchi PS, Schiff S, Ciccola A, Kliegel M. 2009. The role of dual-task and task-switch in prospective memory: Behavioural data and neural correlates. Neuropsychologia. 47(5):1362–1373. 19428400 10.1016/j.neuropsychologia.2009.01.034

[ref6] Brandimonte MA, Ferrante D. 2015. Effects of material and non-material rewards on remembering to do things for others. Front Hum Neurosci. 9:647. 26648861 10.3389/fnhum.2015.00647 PMC4664702

[ref7] Brandimonte MA, Ferrante D, Bianco C, Villani MG. 2010. Memory for pro-social intentions: When competing motives collide. Cognition. 114(3):436–441. 19913218 10.1016/j.cognition.2009.10.011

[ref8] Braver TS, Krug MK, Chiew KS, Kool W, Westbrook JA, Clement NJ, Adcock RA, Barch DM, Botvinick MM, Carver CS, et al. 2014. Mechanisms of motivation–cognition interaction: challenges and opportunities. Cogn Affect Behav Neurosci. 14(2):443–472. 24920442 10.3758/s13415-014-0300-0 PMC4986920

[ref9] Clark-Foos A, Brewer GA, Marsh RL, Meeks JT, Cook GI. 2009. The valence of event-based prospective memory cues or the context in which they occur affects their detection. Am J Psychol. 122(1):89–97. 19353934

[ref10] Cona G, Bisiacchi PS, Moscovitch M. 2014. The effects of focal and nonfocal cues on the neural correlates of prospective memory: insights from ERPs. Cereb Cortex. 24(10):2630–2646. 23645716 10.1093/cercor/bht116

[ref11] Cona G, Kliegel M, Bisiacchi PS. 2015. Differential effects of emotional cues on components of prospective memory: an ERP study. Front Hum Neurosci. 9:10. 25674061 10.3389/fnhum.2015.00010 PMC4309118

[ref12] Cook GI, Rummel J, Dummel S. 2015. Toward an understanding of motivational influences on prospective memory using value-added intentions. Front Hum Neurosci. 9:278. 26042017 10.3389/fnhum.2015.00278 PMC4435068

[ref13] Cousens R, Cutmore T, Wang Y, Wilson J, Chan RC, Shum DH. 2015. Effects of perceptual and semantic cues on ERP modulations associated with prospective memory. Int J Psychophysiol. 98(1):151–156. 26220219 10.1016/j.ijpsycho.2015.07.012

[ref14] Cruz G, Miyakoshi M, Makeig S, Kilborn K, Evans J. 2016. ERPs and their brain sources in perceptual and conceptual prospective memory tasks: Commonalities and differences between the two tasks. Neuropsychologia. 91:173–185. 27520471 10.1016/j.neuropsychologia.2016.08.005 PMC5559711

[ref15] Donchin E, Coles MG. 1988. Is the P300 component a manifestation of context updating. Behav Brain Sci. 11(03):357–427.

[ref16] Einstein GO, McDaniel MA. 1990. Normal aging and prospective memory. J Exp Psychol Learn Mem Cogn. 16(4):717–726. 2142956 10.1037//0278-7393.16.4.717

[ref17] Elliott BL, Blais C, McClure SM, Brewer GA. 2020. Neural correlates underlying the effect of reward value on recognition memory. Neuroimage. 206:116296. 31648002 10.1016/j.neuroimage.2019.116296 PMC8979913

[ref18] Ellis JA. 1996. Prospective memory or the realization of delayed intentions: A conceptual framework for research. In: Brandimonte M, Einstein GO, McDaniel MA, editors. Prospective memory: Theory and applications. Mahwah, NJ: Erlbaum, pp. 115–142.

[ref20] Failing M, Theeuwes J. 2018. Selection history: How reward modulates selectivity of visual attention. Psychon Bull Rev. 25(2):514–538. 28986770 10.3758/s13423-017-1380-y PMC5902518

[ref22] Friedman D, Johnson R Jr. 2000. Event-related potential (ERP) studies of memory encoding and retrieval: A selective review. Microsc Res Tech. 51(1):6–28. 11002349 10.1002/1097-0029(20001001)51:1<6::AID-JEMT2>3.0.CO;2-R

[ref25] Hering A, Phillips LH, Kliegel M. 2014. Importance effects on age differences in performance in event-based prospective memory. Gerontology. 60(1):73–78. 24217232 10.1159/000355057

[ref26] Holm S . 1979. A simple sequentially rejective multiple test procedure. Scand J Statist. 6(2):65–70.

[ref28] Kliegel M, Martin M, McDaniel MA, Einstein GO. 2001. Varying the importance of a prospective memory task: Differential effects across time-and event-based prospective memory. Memory. 9(1):1–11. 11315657 10.1080/09658210042000003

[ref29] Kliegel M, Martin M, McDaniel M, Einstein G. 2004. Importance effects on performance in event-based prospective memory tasks. Memory. 12(5):553–561. 15615314 10.1080/09658210344000099

[ref30] Kok A . 2001. On the utility of P3 amplitude as a measure of processing capacity. Psychophysiology. 38(3):557–577. 11352145 10.1017/s0048577201990559

[ref30a] Krebs RM, Boehler CN, Appelbaum LG, Woldorff MG. 2013. Reward associations reduce behavioral interference by changing the temporal dynamics of conflict processing. PloS one. 8(1):e53894. 10.1371/journal.pone.0053894 PMC3542315 23326530

[ref30b] Locke HS, Braver TS. 2010. Motivational influences on cognitive control: A cognitive neuroscience perspective. Self control in society, mind, and brain. 114–140.

[ref30c] Loft S, Yeo G. 2007. An investigation into the resource requirements of event-based prospective memory. Mem Cognit. 35(2):263–274. 10.3758/bf03193447 17645167

[ref31] Luck SJ . 2014. An introduction to the event-related potential technique. 2nd ed. Cambrigde, MA: MIT Press.

[ref32] Ma DS, Correll J, Wittenbrink B. 2015. The Chicago face database: A free stimulus set of faces and norming data. Behav Res Methods. 47(4):1122–1135. 25582810 10.3758/s13428-014-0532-5

[ref33] MacLean MH, Giesbrecht B. 2015. Neural evidence reveals the rapid effects of reward history on selective attention. Brain Res. 1606:86–94. 25701717 10.1016/j.brainres.2015.02.016

[ref34] Maris E, Oostenveld R. 2007. Nonparametric statistical testing of EEG-and MEG-data. J Neurosci Methods. 164(1):177–190. 17517438 10.1016/j.jneumeth.2007.03.024

[ref35] May C, Owens M, Einstein GO. 2012. The impact of emotion on prospective memory and monitoring: no pain, big gain. Psychon Bull Rev. 19:1165–1171. 22833342 10.3758/s13423-012-0301-3

[ref36] May CP, Manning M, Einstein GO, Becker L, Owens M. 2015. The best of both worlds: emotional cues improve prospective memory execution and reduce repetition errors. Aging Neuropsychol Cogn. 22(3):357–375. 10.1080/13825585.2014.952263 25175608

[ref37] McDaniel MA, Guynn MJ, Einstein GO, Breneiser J. 2004. Cue-focused and reflexive-associative processes in prospective memory retrieval. J Exp Psychol Learn Mem Cogn. 30(3):605–614. 15099129 10.1037/0278-7393.30.3.605

[ref38] Meacham JA, Singer J. 1977. Incentive effects in prospective remembering. J Psychol. 97(2):191–197.

[ref39] Mecklinger A . 2000. Interfacing mind and brain: A neurocognitive model of recognition memory. Psychophysiology. 37(5):565–582. 11037034

[ref40] Meier B, Matter S, Baumann B, Walter S, Koenig T. 2014. From episodic to habitual prospective memory: ERP-evidence for a linear transition. Front Hum Neurosci. 8:489. 25071519 10.3389/fnhum.2014.00489 PMC4079104

[ref41] Nieuwenhuis S, Aston-Jones G, Cohen JD. 2005. Decision making, the P3, and the locus coeruleus--norepinephrine system. Psychol Bull. 131(4):510. 16060800 10.1037/0033-2909.131.4.510

[ref42] Oostenveld R, Fries P, Maris E, Schoffelen JM. 2011. FieldTrip: open source software for advanced analysis of MEG, EEG, and invasive electrophysiological data. Comput Intell Neurosci. 2011:1–9. 21253357 10.1155/2011/156869 PMC3021840

[ref43] Perrin F, Pernier J, Bertrand O, Echallier JF. 1989. Spherical splines for scalp potential and current density mapping. Electroencephalogr Clin Neurophysiol. 72(2):184–187. 2464490 10.1016/0013-4694(89)90180-6

[ref43a] Pessoa L, Engelmann JB. 2010. Embedding reward signals into perception and cognition. Front Neurosci. 4:17. 10.3389/fnins.2010.00017 PMC2940450 20859524

[ref44] Rendell PG, Phillips LH, Henry JD, Brumby-Rendell T, de la Piedad Garcia X, Altgassen M, Kliegel M. 2011. Prospective memory, emotional valence and ageing. Cognit Emot. 25(5):916–925. 21824029 10.1080/02699931.2010.508610

[ref45] Rugg MD, Curran T. 2007. Event-related potentials and recognition memory. Trends Cogn Sci. 11(6):251–257. 17481940 10.1016/j.tics.2007.04.004

[ref46] Rugg MD, Mark RE, Walla P, Schloerscheidt AM, Birch CS, Allan K. 1998. Dissociation of the neural correlates of implicit and explicit memory. Nature. 392(6676):595–598. 9560154 10.1038/33396

[ref47] Rugg MD, Nagy ME. 1989. Event-related potentials and recognition memory for words. Electroencephalogr Clin Neurophysiol. 72(5):395–406. 2469564 10.1016/0013-4694(89)90045-x

[ref48] Rummel J, Smeekens BA, Kane MJ. 2017. Dealing with prospective memory demands while performing an ongoing task: Shared processing, increased on-task focus, or both? J Exp Psychol Learn Mem Cogn. 43(7):1047. 27936845 10.1037/xlm0000359

[ref48a] Sassenhagen J, Draschkow D. 2019. Cluster-based permutation tests of MEG/EEG data do not establish significance of effect latency or location. Psychophysiology. 56(6):e13335. 10.1111/psyp.13335 30657176

[ref49] Schevernels H, Bombeke K, Van der Borght L, Hopf JM, Krebs RM, Boehler CN. 2015. Electrophysiological evidence for the involvement of proactive and reactive control in a rewarded stop-signal task. Neuroimage. 121:115–125. 26188262 10.1016/j.neuroimage.2015.07.023

[ref52] Shelton JT, Scullin MK. 2017. The dynamic interplay between bottom-up and top-down processes supporting prospective remembering. Curr Dir Psychol Sci. 26(4):352–358.

[ref53] Smith RE . 2003. The cost of remembering to remember in event-based prospective memory: investigating the capacity demands of delayed intention performance. J Exp Psychol Learn Mem Cogn. 29(3):347. 12776746 10.1037/0278-7393.29.3.347

[ref55] Smith, R. E., and Hunt, R. R. (2014). Prospective memory in young and older adults: The effects of task importance and ongoing task load. Aging, Neuropsychology, and Cognition. 21(4):411–431. 10.1080/13825585.2013.827150 24628461

[ref57a] Todd Maddox W, Markman AB. 2010. The motivation-cognition interface in learning and decision making. Curr Dir Psychol Sci. 19(2):106–110. 20556228 10.1177/0963721410364008 PMC2885789

[ref59] Wang, Y., Cao, X. Y., Cui, J. F., Shum, D. H., and Chan, R. C. (2013). The relation between prospective memory and working memory: Evidence from event-related potential data. PsyCh journal. 2(2):113–121. 26271181 10.1002/pchj.24

[ref60] West R . 2011. The temporal dynamics of prospective memory: a review of the ERP and prospective memory literature. Neuropsychologia. 49(8):2233–2245. 21187107 10.1016/j.neuropsychologia.2010.12.028

[ref62] West R, Covell E. 2001. Effects of aging on event-related neural activity related to prospective memory. Neuroreport. 12(13):2855–2858. 11588590 10.1097/00001756-200109170-00020

[ref63] West R, Herndon RW, Covell E. 2003. Neural correlates of age-related declines in the formation and realization of delayed intentions. Psychol Aging. 18(3):461. 14518808 10.1037/0882-7974.18.3.461

[ref64] West R, Herndon RW, Crewdson SJ. 2001. Neural activity associated with the realization of a delayed intention. Cogn Brain Res. 12(1):1–9. 10.1016/s0926-6410(01)00014-3 11489603

[ref65] West R, Krompinger J. 2005. Neural correlates of prospective and retrospective memory. Neuropsychologia. 43(3):418–433. 15707617 10.1016/j.neuropsychologia.2004.06.012

[ref65a] West R, Wymbs N. 2004. Is detecting prospective cues the same as selecting targets? An ERP study. Cogn Affect Behav Neurosci. 4(3):354–363. 15535171 10.3758/cabn.4.3.354

[ref66] Wilson J, Cutmore TR, Wang Y, Chan RC, Shum DH. 2013. Effects of cue frequency and repetition on prospective memory: An ERP investigation. Int J Psychophysiol. 90(2):250–257. 23954303 10.1016/j.ijpsycho.2013.08.003

[ref67] Woldorff MG, Liotti M, Seabolt M, Busse L, Lancaster JL, Fox PT. 2002. The temporal dynamics of the effects in occipital cortex of visual-spatial selective attention. Cogn Brain Res. 15(1):1–15. 10.1016/s0926-6410(02)00212-4 12433379

[ref68] Yeung N, Holroyd CB, Cohen JD. 2005. ERP correlates of feedback and reward processing in the presence and absence of response choice. Cereb Cortex. 15(5):535–544. 15319308 10.1093/cercor/bhh153

[ref69] Yeung N, Sanfey AG. 2004. Independent coding of reward magnitude and valence in the human brain. J Neurosci. 24(28):6258–6264. 15254080 10.1523/JNEUROSCI.4537-03.2004 PMC6729539

