# Supplementary material for: The Impact of Error-Consequence Severity on Cue Processing in Importance-Biased Prospective Memory
Source: Cereb Cortex Commun. 2021 Sep 7;2(4):tgab056. doi: 10.1093/texcom/tgab056 (PMC8527855; doi:10.1093/texcom/tgab056)
Supplement: Krasich_CerebralCortex_appendix_tgab056 [file krasich_cerebralcortex_appendix_tgab056.docx]

**Appendix**

*Table A: Findings from the cluster-based permutation analyses*

|  | **Cluster stat** | | **SD** | **95% CI Range** | ***p*_Holm_** |  |
| --- | --- | --- | --- | --- | --- | --- |
| **Not-learned vs. learned controls** |  | |  |  |  |  |
| *Positive clusters* |  | |  |  |  |  |
|  | 3.02 e+4 | | 1.00 e-4 | 1.96 e-4 | .003 | * |
|  | 1.13 e+4 | | 2.64 e-4 | 5.18 e-4 | .016 | * |
|  | 252.50 | | 3.89 e-3 | .01 | 1.000 |  |
|  | 58.67 | | 4.86 e-3 | .01 | 1.000 |  |
|  | 23.75 | | 4.99 e-3 | .01 | 1.000 |  |
|  |  | |  |  |  |  |
| *Negative clusters* |  | |  |  |  |  |
|  | -107.93 | | 4.61 e-3 | .01 | 1.000 |  |
|  | -80.49 | | 4.76 e-3 | .01 | 1.000 |  |
|  | -30.39 | | 4.98 e-3 | .01 | 1.000 |  |
|  |  | |  |  |  |  |
| **PM cues vs. learned controls** |  | |  |  |  |  |
| *Positive clusters* |  | |  |  |  |  |
|  | 5.00 e+4 | | 1.00 e-4 | 1.96 e-4 | .003 | * |
|  | 2.32 e+3 | | 1.60 e-3 | 3.10 e-3 | .454 |  |
|  |  | |  |  |  |  |
| *Negative clusters* |  | |  |  |  |  |
|  | -536.28 | | 3.00 e-3 | .01 | 1.000 |  |
|  | -16.45 | | .01 | .01 | 1.000 |  |
|  |  | |  |  |  |  |
| **Severe vs Moderate PM cues** (correct trials only) | |  | |  |  |  |
| *Positive clusters* |  | |  |  |  |  |
|  | 3.64 e+3 | | 2.00 e+4 | 3.92 e-4 | .010 | * |
|  | 1.13 e+3 | | 1.4 e+3 | 2.70 e-3 | .378 |  |
|  | 18.16 | | 4.9 e+3 | .01 | 1.000 |  |
|  |  | |  |  |  |  |
| *Negative Clusters* |  | |  |  |  |  |
|  | -1.79 e+3 | | 8.40 e-4 | 1.60 e-3 | .156 |  |
|  | -1.31 e+3 | | 1.30 e-3 | 2.60 e-3 | .361 |  |
|  | -609.92 | | 2.60 e-3 | .01 | 1.000 |  |
|  | -127.35 | | 4.70 e-3 | .01 | 1.000 |  |
|  |  | |  |  |  |  |
| **Severe vs Moderate PM cues** (incorrect trials only) | | |  |  |  |  |
| *Positive clusters* | 5.18 e+3 | | 1.00 e-4 | 1.96 e-4 | .003 | * |
|  | 1.52 e+3 | | 1.30 e-3 | 2.5 e-3 | .361 |  |
|  | 567.99 | | 2.80 e-3 | 5.5 e-3 | 1.000 |  |
|  | 195.09 | | 4.30 e-3 | 8.5 e-3 | 1.000 |  |
|  | 79.54 | | 4.90 e-3 | 9.7 e-3 | 1.000 |  |
|  | 26.84 | | 5.00 e-3 | 9.7 e-3 | 1.000 |  |
|  | 13.69 | | 4.80 e-3 | 9.5 e-3 | 1.000 |  |
|  |  | |  |  |  |  |
| *Negative Clusters* |  | |  |  |  |  |
|  | -554.89 | | 2.7 e-3 | 5.4 e-3 | 1.000 |  |

*Note.* Cluster stat = the test statistic that was evaluated under the permutation distribution. The statistic used here was the maximum of the cluster-level statistics; Asterisks (*) indicate significant at *p*_Holm_ < .025.
